# Clinical Profile and Outcome of Snake Bite–Associated Acute Kidney Injury: A Retrospective Study

**DOI:** 10.1155/ijne/6661125

**Published:** 2026-07-15

**Authors:** E. Mahesh, Mohammed Yousuff, N. Monika, Pooja Prabhu, M. S. Gireesh, R. Rajashekar, V. Hamsa

**Affiliations:** ^1^ Department of Nephrology, Ramaiah Medical College and Hospital, Bengaluru, India

**Keywords:** acute kidney injury, antisnake venom, chronic kidney disease, cortical necrosis, snakebite

## Abstract

**Background:**

Snakebite is a neglected tropical disease with a high burden in South Asia, particularly India. Acute kidney injury (AKI) is one of the most serious complications of snake envenomation, which has significant morbidity, mortality, and risk of chronic kidney disease (CKD). The present study aimed to evaluate the incidence, predictors, and outcomes of snakebite‐associated AKI (SBE‐AKI) in a tertiary care center.

**Methods:**

We retrospectively analyzed 325 patients with snakebite envenomation, admitted to our institution. Demographic, clinical, laboratory, and treatment variables were compared between patients with and without AKI. AKI was staged according to KDIGO criteria. Renal biopsy was performed selectively in patients with prolonged renal failure, dialysis dependence, delayed renal recovery, or suspicion of irreversible renal injury. Outcomes assessed included recovery, progression to CKD, and mortality.

**Results:**

Of the 325 patients, 79 (32.1%) developed AKI. Patients with AKI were significantly younger (mean age 34 vs. 45 years, *p* = 0.001). Delay in antisnake venom (ASV) administration (5 vs. 9 h, *p* = 0.001), need for inotropes (41.8% vs. 14.2%, *p* = 0.001), and mechanical ventilation (36.7% vs. 6.9%, *p* = 0.001) were strong predictors. Proteinuria was more frequent in AKI (80% vs. 32.5%). Among AKI patients, 57% had Stage 3 AKI; 39.2% required dialysis. Biopsy (*n* = 8) showed acute tubular necrosis in 37.5% and cortical necrosis in 25%. Outcomes included 77.2% recovery, 6.3% progression to CKD, and 16.5% mortality.

**Conclusion:**

SBE‐AKI is a common and serious complication of snakebite. Delay in ASV administration, hemodynamic instability, proteinuria, advanced AKI stage, and cortical necrosis predict poor outcomes. Early ASV, timely dialysis, and long‐term nephrology follow‐up are essential to improve survival and reduce CKD progression.

## 1. Introduction

Snakebite envenomation is a major yet neglected tropical disease, affecting rural and agricultural communities in Asia, Africa, and Latin America. The World Health Organization (WHO) estimates that 4.5–5.4 million people are bitten annually worldwide, resulting in 1.8–2.7 million cases of envenomation and 81,000–138,000 deaths each year [1]. India contributes nearly half of this global burden, with an estimated 58,000 deaths annually, accounting for up to 0.5% of all deaths in the country [2]. Snakebite is thus not only a medical emergency but also a significant public health and occupational hazard in developing nations.

Acute kidney injury (AKI) is one of the most serious systemic complications of snakebite. The incidence of snakebite‐associated AKI (SBE‐AKI) varies widely, ranging from 13% to 32% in India and up to 45% in Southeast Asia, depending on the offending species, geographic distribution, and timeliness of treatment [3–5]. Mortality in patients with SBE‐AKI remains high, between 20% and 50% in earlier studies [6]. Moreover, even among survivors, there is an appreciable risk of progression to chronic kidney disease (CKD), thereby contributing to the global CKD burden [7].

The pathogenesis of SBE‐AKI is multifactorial. Based on previously described mechanisms of common Indian snake envenomation syndromes, hemotoxic and myotoxic venoms can cause systemic and renal injury via multiple mechanisms, including direct nephrotoxicity of venom components (phospholipase A2, metalloproteinases, and serine proteases), hemodynamic instability from shock and capillary leak, and coagulopathy and thrombotic microangiopathy, leading to cortical necrosis.

Pigment nephropathy due to hemoglobinuria and myoglobinuria from intravascular hemolysis and rhabdomyolysis, secondary sepsis, and local tissue necrosis, further aggravate renal insult [6, 8].

Histologically, the renal lesions range from acute tubular necrosis (ATN), the most frequent and potentially reversible lesion, to acute interstitial nephritis (AIN) and diffuse cortical necrosis, which is associated with irreversible renal failure [9, 10].

Access to antisnake venom (ASV) and renal replacement therapy (RRT) are central to reducing mortality [8]. Unfortunately, in rural India and other resource limited regions, delays in ASV administration and limited availability of dialysis often lead to poor outcomes [3–5].

Given this background, the present study was undertaken to evaluate the clinical profile, predictors, and outcomes of SBE‐AKI in a tertiary care setting.

## 2. Objectives

1. To evaluate the clinical profile of snakebite patients.
2. To determine predictors of developing AKI following snakebite.
3. To assess outcomes of patients with snakebite‐related AKI.

## 3. Methodology

This was a retrospective observational study conducted at hospitals attached to Ramaiah University of Applied Sciences, Bangalore, between January 2013 and December 2023. All patients with history of snakebite were included. AKI was diagnosed and staged according to KDIGO guidelines. As pre‐envenomation baseline serum creatinine values were unavailable in most patients due to the retrospective nature of the study, baseline renal function was estimated using the lowest recorded serum creatinine during hospitalization in patients who recovered renal function. Urine output criteria were additionally used where appropriate. Patients were grouped into AKI and non‐AKI cohorts. Clinical, laboratory, and outcome parameters were compared between groups.

Renal biopsy was performed selectively in patients with prolonged renal failure, dialysis dependence, delayed renal recovery, or suspicion of irreversible renal injury.

## 4. Statistical Analysis

Data entry was performed using Microsoft Excel, and statistical analysis was conducted using SPSS Version 21. Continuous variables were assessed for normality using the Shapiro–Wilk test. Normally distributed variables were expressed as mean ± standard deviation (SD), while skewed variables were expressed as median with interquartile range (IQR). Categorical variables were summarized as frequencies and percentages. Comparisons between AKI and non‐AKI groups were performed using the independent sample *t*‐test for normally distributed continuous variables and the Mann–Whitney *U* test for nonnormally distributed variables. Chi‐square test or Fisher’s exact test was used for categorical variables. Multivariable regression analysis was performed to identify predictors of AKI severity, dialysis requirement, and mortality. A *p* value < 0.05 was considered statistically significant.

Descriptive statistics of the explanatory and outcome variables were calculated by mean, SD for quantitative variables, frequency, and proportions for qualitative variables.

Inferential statistics like the chi‐square test was applied for qualitative variables to find the association. The independent sample *t* test was applied to compare the quantitative parameters between the groups. The level of significance was set at 5%.

## 5. Results

A total of 325 patients with snakebite were included in the study, of whom 79 (32.1%) developed AKI.

### 5.1. Comparison of Demographic, Clinical, Laboratory Parameters, AKI Characteristics, and Outcomes (Table 1)

#### 5.1.1. Demographic Characteristics

The mean age of patients in the AKI group was significantly lower than that in the non‐AKI group with male preponderance (34.2 ± 17.4 vs. 45.3 ± 16.6 years, *p* = 0.001). Hypertension was significantly more frequent among AKI patients (13.9% vs. 6.5%, *p* = 0.038), while diabetes mellitus showed a nonsignificant trend toward higher prevalence in AKI patients (12.7% vs. 6.1%, *p* = 0.057).

**TABLE 1:** Comparison of demographic, clinical, laboratory parameters, AKI characteristics, and outcomes.

Parameter	Non‐AKI	AKI	*p* value
Age (years)	45.34 ± 16.6	34.17 ± 17.3	0.001^∗^
Male sex	185 (75.2%)	60 (75.9%)	0.893
Diabetes mellitus	15 (6.1%)	10 (12.7%)	0.057
Hypertension	16 (6.5%)	11 (13.9%)	0.038^∗^
Requirement of inotropes	35 (14.2%)	33 (41.8%)	0.001^∗^
Mechanical ventilation	17 (6.9%)	29 (36.7%)	0.001^∗^
Anuria	1 (0.4%)	10 (12.7%)	0.001^∗^
Oliguria	0	26 (32.9%)	0.001^∗^
Time to ASV administration	5 (4–7)	9 (5.75–24)	0.001^∗^
Total ASV dose	12.8 ± 10.6	20.4 ± 7.7	0.001^∗^
Hospital stay	4 (3–5)	8 (5–15)	0.001^∗^
Serum urea (mg/dL)	12.59 ± 6.58	48.62 ± 39.03	0.001^∗^
Serum creatinine Day 1 (mg/dL)	0.92 ± 0.22	4.26 ± 3.43	0.001^∗^
Hemoglobin (g/dL)	14.43 ± 2.16	11.64 ± 3.25	0.001^∗^
Total leukocyte count	11,946 ± 5444	16,499 ± 8017	0.001^∗^
INR	1.69 ± 1.57	2.18 ± 2.46	0.038^∗^
Proteinuria present	79 (32.5%)	63 (80%)	0.001^∗^
Compartment syndrome	35 (14.2%)	30 (38%)	0.001^∗^
Fasciotomy	14 (5.7%)	23 (29.1%)	0.001^∗^
Cellulitis	56 (22.8%)	43 (54.4%)	0.001^∗^
Stage 1 AKI	—	23 (29.1%)	—
Stage 2 AKI	—	11 (13.9%)	—
Stage 3 AKI	—	45 (57%)	—
RRT requirement	—	31 (39.2%)	—
Biopsy‐proven ATN	—	3 (37.5%)	—
Cortical necrosis	—	2 (25%)	—
AKI recovery	—	61 (77.2%)	—
Progression to CKD	—	5 (6.3%)	—
Mortality	—	13 (16.5%)	—

^∗^Indicates statistically significant.

#### 5.1.2. Clinical Parameters

Patients who developed AKI had a significantly higher requirement for inotropes (41.8% vs. 14.2%, *p* = 0.001) and mechanical ventilation (36.7% vs. 6.9%, *p* = 0.001) compared with the non‐AKI group. Urine output at presentation differed markedly: oliguria was seen in 32.9% and anuria in 12.7% of AKI patients, whereas almost all non‐AKI patients had normal urine output (*p* = 0.001).

The time interval from snakebite to ASV administration was significantly longer in the AKI group and showed a skewed distribution; therefore, it has been expressed as median with IQR. Delayed ASV administration was significantly associated with AKI development (*p* = 0.001). Median duration of hospital stay was significantly longer in AKI patients (8 [5–15] days) compared with the non‐AKI group (4 [3–5] days, *p* = 0.001).

#### 5.1.3. Laboratory Findings

Patients with AKI had markedly elevated serum urea (48.6 ± 39.0 vs. 12.6 ± 6.6 mg/dL, *p* = 0.001) and serum creatinine at Day 1 (4.26 ± 3.43 vs. 0.92 ± 0.22 mg/dL, *p* = 0.001). Anemia was significantly more common in the AKI group (Hb 11.6 ± 3.3 vs. 14.4 ± 2.2 g/dL, *p* = 0.001) as was leukocytosis (TLC 16,499 ± 8017 vs. 11,947 ± 5444 cells/mm^3^, *p* = 0.001). INR values were also higher in the AKI group (2.18 ± 2.46 vs. 1.69 ± 1.57, *p* = 0.038).

Urinalysis abnormalities (proteinuria) were significantly more frequent among AKI patients (80% vs. 32.5%, *p* = 0.001).

#### 5.1.4. Complications and Interventions

Compartment syndrome occurred in 38% of the AKI patients compared with 14.2% of the non‐AKI patients (*p* = 0.001). Surgical interventions such as fasciotomy (29.1% vs. 5.7%) and debridement (25.3% vs. 16.3%) were more common in the AKI group. Infective complications were also significantly higher in AKI patients, with cellulitis in 54.4% (vs. 22.8% in non‐AKI), necrotizing fasciitis in 3.8% (vs. 0.4%), and cases of wet gangrene and DIC with MODS occurring exclusively in the AKI group.

Within the AKI cohort, Stage 3 AKI was most common (57%), followed by Stage 1 (29.1%) and Stage 2 (13.9%). RRT was required in 39.2% of the AKI patients, predominantly hemodialysis.

Renal biopsy was performed in 8 patients: ATN was the most frequent lesion (37.5%), followed by acute on chronic interstitial nephritis, and complete cortical necrosis (25%).

##### 5.1.4.1. Outcomes

Of the AKI patients, 77.2% recovered renal function completely, 6.3% progressed to CKD, and 16.5% succumbed to illness. Mortality was strongly associated with Stage 3 AKI, anuria, shock, and delayed ASV administration.

**FIGURE 1:**
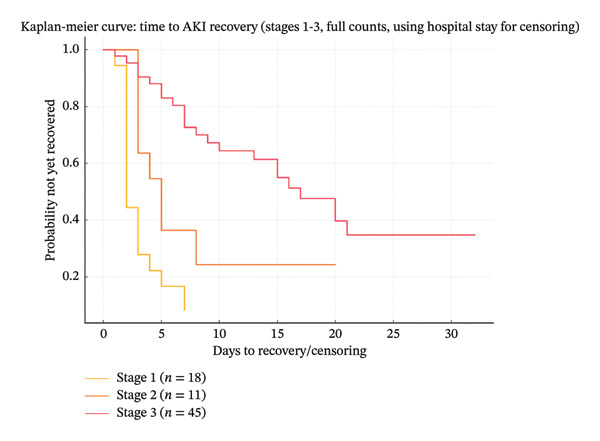
Kaplan–Meier curve showing time to AKI recovery.

Kaplan–Meier curve (Figure 1) shows the trend in recovery of AKI based on AKI severity. Rapid recovery was seen in Stage 1 AKI where recovery was seen by 5–7 days. AKI Stage 3 shows longest recovery time, indicating more severe injury with delayed renal recovery.

Predictors of AKI severity (ordinal logistic regression): Every hour delay increases odds of more severe AKI by **4.2%.** Not requiring ventilation reduces odds of severe AKI by 83%.

Predictors of dialysis requirement (binary logistic regression): Delay in ASV administration and requirement of mechanical ventilation were independently associated with dialysis requirement.

Predictors of mortality (multinomial logistic regression) (Table 2): Mechanical ventilation is the single strongest predictor of death in snakebite AKI.

**TABLE 2:** Multivariate regression.

Predictor	AKI severity (ordinal) OR (95% CI)	*p* value	Dialysis requirement (binary) OR (95% CI)	*p* value	Death vs. recovery (multinomial) OR (95% CI)	*p* value
Age (per year)	1.00 (0.97–1.03)	0.989	**1.05 (1.01–1.10)**	**0.026** ^∗^	0.98 (0.93–1.04)	0.456
ASV delay (per hour)	**1.04 (1.01–1.08)**	**0.016** ^∗^	**1.06 (1.02–1.09)**	**0.002** ^∗^	1.02 (0.97–1.06)	0.490
ASV dose	0.98 (0.91–1.05)	0.516	0.99 (0.80–1.20)	0.753	1.07 (0.96–1.19)	0.244
Male sex	1.02 (0.30–3.00)	0.972	0.93 (0.25–3.40)	0.920	0.30 (0.04–2.10)	0.225
Diabetes (absent)	5.00 (0.92–10.0)	0.063	7.68 (0.92–12.0)	0.062	3.66 (0.18–73.8)	0.397
Hypertension (absent)	0.25 (0.05–1.50)	0.124	0.95 (0.05–1.50)	0.959	0.14 (0.01–1.91)	0.139
Inotropes (No)	0.97 (0.30–1.80)	0.957	1.63 (0.50–3.50)	0.497	0.27 (0.04–1.69)	0.161
Mechanical ventilation (No)	**0.17 (0.05–0.61)**	**0.007** ^∗^	**0.08 (0.02–0.33)**	**0.001** ^∗^	**0.08 (0.01–0.58)**	**0.013** ^∗^

*Note:* Bold values indicate statistically significant.

^∗^Indicates statistically significant.

## 6. Discussion

The majority of patients in our study were young males, consistent with the occupational risk of farming and outdoor activities. Male predominance has been consistently reported across Indian studies [4, 5]. The mean age was significantly lower in AKI patients (34 years) compared with non‐AKI (45 years), reinforcing that snakebite nephropathy primarily affects healthy, economically active populations.

Pre‐existing hypertension was significantly more common in the AKI group, and diabetes showed a trend toward higher prevalence. While not traditionally considered risk factors in envenomation, these comorbidities may reduce renal reserve, thereby amplifying the impact of venom‐induced ischemia and coagulopathy.

Clinically, the need for inotropes and mechanical ventilation correlated strongly with AKI, underscoring that the systemic severity of envenomation predicts renal outcomes. The high prevalence of severe systemic manifestations in our cohort likely reflects referral bias inherent to a tertiary care nephrology center, where patients with complicated snakebite envenomation are preferentially referred after initial stabilization elsewhere. Reduced urine output (oliguria/anuria) was an early clinical sign of impending renal failure, consistent with previous reports [8].

A critical finding in our study was the delay in ASV administration among patients who developed AKI. The median time from snakebite to ASV administration was significantly longer in the AKI group (9 [5.75–24] hours) compared with the non‐AKI group (5 [4–7] hours), highlighting the time‐sensitive nature of antivenom therapy. Delayed administration of ASV allows prolonged circulation of venom toxins, thereby increasing the risk of systemic complications including coagulopathy, shock, and renal injury. Previous studies have similarly demonstrated that the early administration of ASV, preferably within the first few hours following envenomation, is associated with lower incidence of AKI and improved survival outcomes [8, 11]. Our findings reinforce the importance of early referral, rapid transport, and timely access to ASV, particularly in rural and resource‐limited settings where delays in treatment remain common.

Additionally, AKI patients required a higher total ASV dose (20 vials vs. 13 vials), likely reflecting greater venom load and severity of envenomation.

Our AKI cohort demonstrated classical biochemical derangements: markedly elevated serum urea and creatinine, along with significant anemia, leukocytosis, and prolonged INR. These reflect hemolysis, systemic inflammation, and venom‐induced coagulopathy.

A notable observation was the strong association between AKI and urinary abnormalities, present in 80% of the AKI patients vs. 32.5% of non‐AKI. Proteinuria suggests glomerular barrier injury, while hematuria may arise from both glomerular and tubular lesions. Prior studies [9, 10] have correlated these findings with renal biopsy features such as ATN, interstitial nephritis, and cortical necrosis. Importantly, urinalysis is a simple, low‐cost bedside tool that may help in early risk stratification, even before overt azotemia develops.

Using KDIGO criteria, Stage 3 AKI was the most common (57%), followed by Stage 1 (29%) and Stage 2 (14%). This indicates that most patients present late with advanced renal injury, often necessitating dialysis. In our cohort, nearly 40% required RRT, similar to other Indian studies [6, 8].

Staging also predicted outcomes: patients with Stage 1 AKI had higher rates of recovery, while Stage 3 was strongly associated with mortality and CKD progression.

Renal biopsy, though performed in a small subset, provided valuable insights. ATN was the predominant lesion, confirming it as the morphological hallmark of snakebite AKI [9, 10]. ATN results from a combination of ischemia, pigment nephropathy (hemoglobin/myoglobin), and direct tubular cytotoxicity of venom enzymes such as phospholipase A2.

Cortical necrosis, observed in 25% of the biopsied patients, is less common but carries grave implications, usually leading to irreversible renal failure. AIN was also noted, reflecting immune‐mediated injury or hypersensitivity to venom proteins. Importantly, AIN may respond to corticosteroid therapy, suggesting a potential therapeutic role for early renal biopsy in select patients.

Encouragingly, 77.2% of the patients with AKI achieved complete renal recovery. However, 16.5% died and 6.3% progressed to CKD. The mortality observed in our cohort was lower than that reported in earlier Indian studies, which documented mortality rates ranging from 20% to 50%, possibly reflecting improved access to dialysis and critical care support in recent years [6, 9]. Mortality was strongly associated with Stage 3 AKI, anuria, shock, and delayed administration of ASV. Progression to CKD occurred predominantly in patients with cortical necrosis or prolonged dialysis dependence, highlighting the importance of long‐term renal follow‐up in survivors of severe SBE‐AKI [11, 12].

This finding aligns with the emerging concept of the AKI‐to‐CKD continuum, where survivors of severe AKI remain at risk of long‐term renal impairment [12]. Snakebite nephropathy, therefore, contributes not only to acute mortality but also to the burden of CKD in endemic regions [7].

Our findings align with prior Indian and Southeast Asian studies [5, 6, 8], [10, 12], which emphasize (1) early ASV as the cornerstone of prevention. (2) ATN as the commonest lesion, cortical necrosis as the worst prognosis. (3) High recovery rates if dialysis is available. (4) Significant risk of CKD in survivors.

Across all multivariable models, time from snakebite to ASV administration and requirement of mechanical ventilation emerged as the strongest and most consistent predictors of adverse renal outcomes. Delay in ASV increased the odds of severe AKI and dialysis requirement, while the need for mechanical ventilation reflecting systemic envenomation severity was the most powerful predictor of both AKI severity and mortality. Age mildly increased dialysis risk. No significant associations were found with sex, diabetes, hypertension, or total ASV dose.

## 7. Conclusion

Snakebite is an important and preventable cause of AKI in tropical countries, disproportionately affecting young rural populations. Delay in ASV administration, presence of shock and respiratory failure, oliguria/anuria, and severe local complications were significant predictors of AKI.

While most patients recovered, cortical necrosis and severe AKI led to CKD progression or death in a significant proportion. Early ASV administration, dialysis availability, and long‐term follow‐up are essential to reduce morbidity and mortality from SBE‐AKI.

### 7.1. Limitations

This was a single‐center study.

Renal biopsy was performed in only a small proportion of patients, and biopsy findings cannot be generalized to the entire cohort. The offending snake species could not be identified in most patients; therefore, species‐specific analysis was not possible.

There was no standardized protocol for antivenom (ASV) dosing, and long‐term follow‐up data on CKD outcomes were unavailable for all patients.

## Funding

No funding was received for this manuscript.

## Disclosure

The manuscript was submitted in arxiv as a preprint screening. It has not been published in any journal [13].

## Conflicts of Interest

The authors declare no conflicts of interest.

## Data Availability

The data that support the findings of this study are available from the corresponding author upon reasonable request.
